# Chitin Extracted from Black Soldier Fly Larvae at Different Growth Stages

**DOI:** 10.3390/polym16202861

**Published:** 2024-10-10

**Authors:** Andrea Marangon, Geo Paul, Riccardo Zaghi, Leonardo Marchese, Giorgio Gatti

**Affiliations:** 1 Dipartimento per lo Sviluppo Sostenibile e la Transizione Ecologica, Università degli Studi del Piemonte Orientale, Piazza S. Eusebio 5, 13100 Vercelli, Italy; andrea.marangon@uniupo.it; 2 Dipartimento di Scienze e Innovazione Tecnologica, Università degli Studi del Piemonte Orientale, Viale Teresa Michel 11, 15121 Alessandria, Italy; geo.paul@uniupo.it (G.P.); leonardo.marchese@uniupo.it (L.M.); 3 Myia SA, Via Industria 12, CH-6710, 6710 Biasca, Switzerland; riccardo.zaghi@myiasa.ch

**Keywords:** chitin characterization, chitin from insect, extraction from waste materials, black soldier fly, X-ray diffractometry, infrared spectroscopy, ^13^C CPMAS NMR, thermogravimetric analysis

## Abstract

The black soldier fly (BSF) *Hermetia Illucens* can grow rapidly and on a wide variety of organic materials, and it is extensively used as a means of disposing of household organic waste. Different phases of the life cycle of BSF larvae (BSFL) are used in this work to extract chitin after the removal of lipids, mineral salts, and proteins. Multiple techniques, such as X-ray diffractometry, infrared spectroscopy, solid-state Nuclear Magnetic Resonance (^13^C ss-NMR) and thermogravimetric analysis, are used to investigate the chemical and physical characteristics of the extracted samples of chitin, which shows a high degree of acetylation (from 78% to 94%). The extracted chitin shows an increase of the thermal stability of 20 °C in the initial stage of life and 35 °C at the end of the life cycle if compared with a commercial standard. Moreover, the extracted chitin shows an increase in the crystallinity degree during the BSFL growth time (from 72% to 78%).

## 1. Introduction

Insect farms are one of the most emerging and sustainable industrial activities, and during the last years, several insect species have been bred to produce animal feed and fertilizers for agricultural use or for processing organic waste [1]. The use of different insect species changes the ended products: among these, organic waste processing Black Soldier Fly Larvae (BSFL), a promising technology with high waste reduction potential, leads to marketable high-value products [1,2,3,4].

BSFL are originally native to the Americas and widespread in tropical and temperate regions of the world; the species shows a short life cycle of 41 to 131 days, depending on the nutrient and energy composition of the feeding substrates and the ambient temperature of the rearing environment [5,6]. The short life cycle and fast growth potential of BSFL make this species interesting for farming conditions: depending on the moisture content of the substrate, BSFL can consume daily 2 to 6.5 times their body mass in feed [7,8,9,10,11,12,13,14].

Among the products that can be obtained by BSFL processing, resources for livestock feed can be mentioned. The Dry Matter (DM) content of fresh BSFL is high (34.9% to 44.9%), and this makes BSFL less expensive than other fresh products. On average, BSFL consist of 41 to 44% crude protein (CP), 15 to 35% ethereal extract (EE), 7 to 10% crude fibers (CF), 15 to 28% ash and about 5.250 kcal/kg of gross energy (GE), based on DM [8]. Moreover, BSFL are high in Ca (5 to 8%) and P (0.6 to 1.5%) [8].

The fraction of protein, lipids and mineral salts depends on various environmental factors during the growth of the larvae, ranging from the conditions under which BSFL are reared to the processing methods they undergo for transformation into final products [9,15,16,17]. BSFL have been also used as a source of new antibiotic preparations for livestock, as well as a source of chitin and its derivative chitosan [12,13]. Recently, their use in the generation of energy in the form of biogas through the anaerobic digestion of frass has also been explored [11].

**Figure 1:**
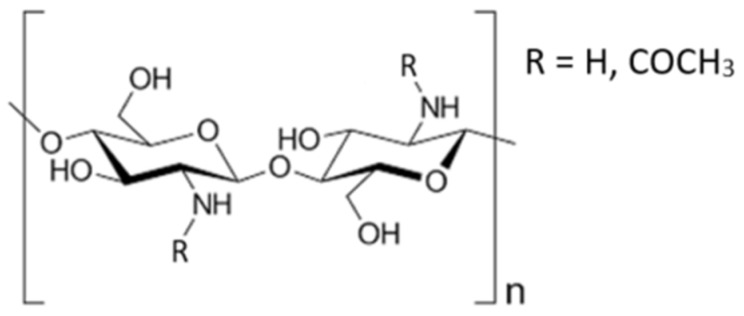
Schematic representation of both chitin, where R = COCH_3_ (acetyl group) is greater than 60%, and chitosan, where R = H is greater than 60%.

Chitin has a cellulose structure (Figure 1) in which the hydroxyl group at the carbon atom in position 2 is replaced by an acetamide group. It is one of the most widely occurring biopolymers in nature, only second to cellulose, and consists of N-acetyl-2-amino-2-deoxy-D-glucopyranose and 2-amino-2-deoxy-D-glucose in pyranose form, which are linked together by 1–4 glycosidic bonds with a degree of acetylation (DA%) greater than 60 percent [18,19,20].

It is naturally present in the exoskeletons of various animals, fungi, and microorganisms. Currently, the main source from which chitin is extracted is seafood industry waste, particularly from the exoskeletons of crabs and shrimps [2,18,21,22].

Chitin is widely used in various fields, such as in biomedical applications due to its biocompatibility and ability to reabsorb into tissues, in pharmacology as a carrier, in agriculture and water treatment as an adsorbent or flocculant, and in the textile and food industries as a stabilizer. The application of chitin in these different fields is due to its properties as an adsorbent and antimicrobial [2,18,19,23,24,25,26]. Chitin and chitosan-derived composites offer biocompatibility, excellent barrier and mechanical properties, and are cost-effective compared to synthetic materials. In food packaging, they act as a carrier for antioxidants and antimicrobial agents, enhancing food preservation and extending shelf life, making them ideal for sustainable packaging solutions [27]. Besides insects, fungi and microorganisms also contain chitin, though in a much lower percentage than in the exoskeletons of arthropods [28].

Extracting chitin from insects has several advantages because they (i) are not subject to seasonality as in the case of fisheries, (ii) can survive in suboptimal conditions, and (iii) require less space and water than other sources. In addition, insects have a shorter life cycle when compared to other sources [2,3].

Chitosan is also present in nature, though in a form with a lower degree of acetylation (<60%). Figure 1 shows a schematic representation of chitin and chitosan.

As a natural biopolymer, chitin is found in three allomorphic forms and with varying degrees of acetylation: (i) α-chitin is the most abundant form in the exoskeleton of crustaceans, and this type of chitin chain has an antiparallel structure; (ii) β-chitin extracted from squid pens is characterized by the parallel chitin chain structure and (iii) γ-chitin, the less abundant form, is found in yeast and fungi as a combination of α-chitin and β-chitin. Crustaceans, fungi, and insects have different allomorphic forms of chitin with varying degrees of acetylation [2,19,21,22].

The process of isolating chitin from BSFL was optimized in this work in terms of temperature, time and the concentration of reagent. The extracted chitin was compared with a commercial one and the difference in terms of the acetylation degree and crystallinity were studied. The chitin obtained from BSFL shows a high degree of acetylation (DA%) (some set samples at more than 90%) if compared with the highest chitin extract obtained from insects reported in the literature.

## 2. Materials and Methods

### 2.1. Materials

The materials from which chitin was extracted were samples from different BSFL stages. The sets of samples were collected at 10 days, in the initial phase of BSFL life (sample named BSFL-IL), and 25 days, in the end phase of BSFL life (sample named BSFL-EL) after hatching, respectively. All samples used were derived from a Myia SA (Biasca, Switzerland) pilot plant in which both colony reproduction for egg production and larval fattening on organic substrates for the final production of feed materials were carried out. The larvae used in the experiment were grown on UFA 524 IPS chicken starter feed prepared at a 75% moisture content. The inoculum of 5-DOL (Days Of Larvae) was calculated at the rate of 1.5 g feed/5-DOL.

The chitin extracted from each sample was compared with a commercial sample of chitin (TCI chemicals (Tokyo, Japan), CAS number 1398-61-4).

### 2.2. Chitin Extraction and Purification

Black Soldier Fly Larvae samples were dried at 50 °C overnight to remove the water contained within the samples. After the removal of water from 1 g of sample, the first process step was carried out for the extraction of lipids with 50 mL of dichloroethane (Sigma-Aldrich (St. Luois, MO, USA), CAS number 107-06-2), and the sample was stirred for 24 h. This process is needed because lipids can interfere with the subsequent steps. The materials were treated with 1 M HCl (Sigma-Aldrich (St. Luois, MO, USA), CAS number 7647-01-0), with a sample to solution ratio of 1:50, and heated at 95 °C for one hour. This step provides the solubilization of the carbonate salts contained in the materials [29]. After one hour, the samples were filtered and washed with deionized water to obtain a neutral pH and then dried at 50 °C up to constant weight. Subsequently, the demineralized samples were placed in contact with 1 M NaOH (Sigma-Aldrich (St. Luois, MO, USA), CAS number 1310-73-2), with a sample to solution ratio of 1:50. The suspensions were heated at boiling point for 24 h. This step was conducted with the aim of solubilizing the protein fraction and leaving the chitin present unaltered [29]. After 24 h, the materials were separated from the solution by filtration and washed with deionized water to a neutral pH, after which the samples were dried at 50 °C up to constant weight. The percentages of removed substances, for each step, are reported in Table 1, which shows the composition of two sets of BSFL samples (BSFL-IL and BSFL-EL).

### 2.3. Characterization Techniques

X-ray powder diffraction (XRPD) patterns were obtained using a AXS D8 ADVANCE diffractometer (Bruker, Billerica, MA, USA) in reflection mode with Bragg–Brentano geometry, using an interval of 5–40° (2 θ), operating with a radiation source of monochromatic X-rays at Cu Kα (λ = 1.5406 Å), using a LYNXEYE_XE_T detector, and applying 40 kV and 40 mA as the voltage and amperage, respectively.

Attenuated Total Reflectance infrared spectroscopy (ATR-FTIR) analyses were carried out by using an IR Nicolet 5700 (Thermo Fisher Scientific, Waltham, MA, USA) spectrometer with 64 scans and a resolution of 4 cm^−1^ in a 4000–400 cm^−1^ spectral range. Solid-state NMR spectra were collected using a Bruker Avance III 500 spectrometer (Bruker, Billerica, MA, USA) and a wide-bore 11.75 Tesla magnet, with operational frequencies of 125.77 MHz for C. In all experiments, a 4 mm triple resonance probe, operating in double resonance mode with magic angle spinning (MAS), was utilized. Samples were loaded into a 4 mm ZrO_2_ rotor and spun at a MAS rate of 10–15 kHz. For the ^13^C cross-polarization (CP) MAS experiments, initial excitation and decoupling were achieved using proton radio frequencies (RF) of 55 and 28 kHz, respectively. Throughout the CP period, the ^1^H RF field underwent ramping in 100 increments, while the ^13^C RF field remained constant. Protons were decoupled from carbon during acquisition using a Spinal-64 decoupling scheme. A cross-polarization contact time of 2 ms and a delay between scans of 1 to 5 s were employed [30]. Spin locking utilized a moderate ramped RF field of 55 kHz, and the carbon RF field was adjusted for optimal signal (40 kHz). Spectra were recorded with a spectral width of 42 kHz, and transients between 5 k and 40 k were accumulated at 298 K. Chemical shifts are reported using the δ scale and externally referenced to TMS at 0 ppm.

Thermogravimetry analyses (TGA) were performed on a Setaram (Caluire, France) LABSYS evo (TGA, DTA/DSC) under N_2_ (gas flow rate 40 mL/min), heating around 10 mg of samples from 30 to 700 °C at a rate of 5 °C/min [31].

## 3. Results

Chitin was extracted by adapting methods used for the extraction of chitin and chitosan from crab and shrimp exoskeletons [2,3,21,27,28]. Several methods for the extraction of chitin from insects are described in the literature, with each process differentiated by the time, acid or base concentration, operating temperature, and order of demineralization and deproteinization process [32,33,34,35,36]. Chitin can be extract from different sources with chemical, biological, or enzymatic processes [22,36,37,38]. In this work, a chemical extraction process was used for chitin extraction from BSFL.

After removing lipids, mineral salts, and proteins, all samples were analyzed using different techniques to determine the composition of the obtained materials (Table 1). Each step was repeated five times on different batches with the aim of obtaining an average value.

### 3.1. Characterisation

Infrared spectroscopy was used to characterize the untreated materials, as well as those after each processing stage. The extracted chitin samples were subjected to infrared spectroscopy, thermogravimetric analysis, X-ray diffractometry and ^13^C CPMAS NMR. On the extracted chitin samples, infrared spectroscopy, thermogravimetric analysis, X-ray diffractometry and ^13^C CPMASNMR analyses were carried out. Such extended characterization aimed to determine the differences in the materials after the process of protein extraction. The chitin samples obtained from the BSFL samples at different growing times were extracted using the methods described in this work and compared to a commercial chitin.

### 3.2. X-ray Diffraction

The X-ray diffraction (XRD) analysis of chitin samples is widely used, as it enables the determination of the degree of crystallinity [19,20,28,39].

**Figure 2:**
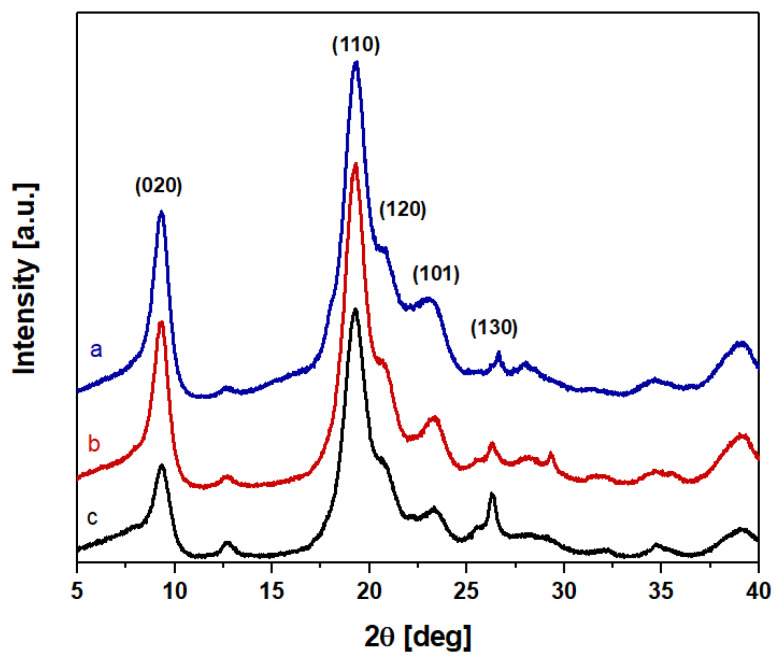
XRD of BSFL samples at the start and at the end of their life cycle; BSFL-IL (a) and BSFL-EL (b), respectively, compared with a commercial chitin (c).

The XRD profiles of BSFL-IL and BSFL-EL, in Figure 2 a and b, respectively, show the characteristic diffraction peaks of chitin, though some of them, ranging between 25 and 30 2θ, could not be identified due to the presence of residual impurities of mineral salts and proteins. The chitin extracted from the BSFL, both at the start and end of the life cycle, shows the same XRD profile as the commercial chitin. The presence of impurities or residual protein causes the widening of reflexes and a small shift in the peaks’ position. Previous research was used to assign the reflection planes in the diffractograms [19,39,40].

No significant differences in the position of the reflection peaks were detected; the intensity of the peaks was used to determine the Cristal Index of the extracted chitin samples and the commercial one.

From the diffractograms, the Crystal Index (CrI%) was calculated using Equation (1) [41]:

(1) $$CrI\%=\frac{I_{020}-I_{am}}{I_{020}}\cdot 100$$

where I_020_ is the intensity of the peak attributed to the 020 plane (2θ ≈ 9°) and I_am_ is the intensity of the amorphous contribution present in the diffractogram at 2θ ≈ 16° [42]. Table 2 shows the CrI% (average of five replications) of the extracted chitin samples.

### 3.3. FT-IR Spectroscopy

**Figure 3:**
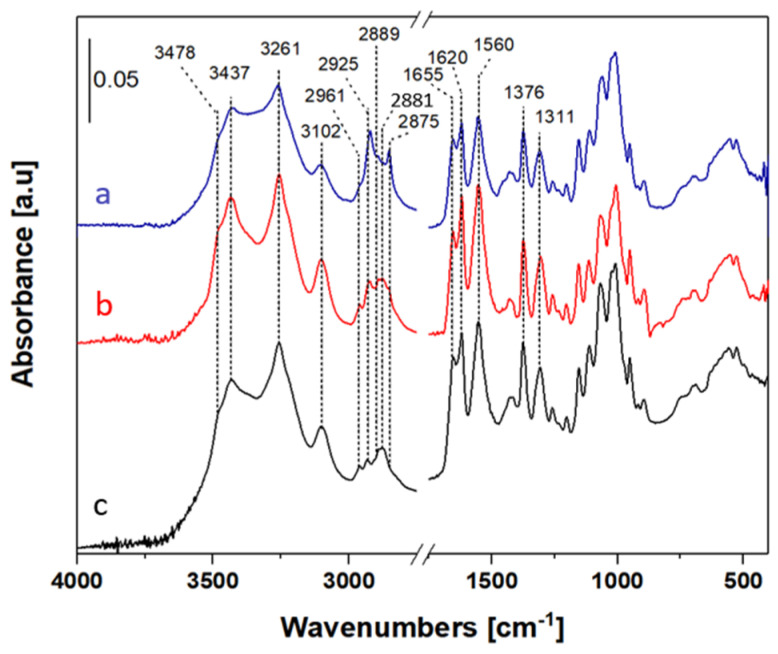
ATR-FTIR spectra of chitin samples extracted from BSFL at the start and at the end of their life cycle: BSFL-IL (a) and BSFL-EL (b), respectively. The spectrum of a commercial chitin (c) is reported for comparison.

Attenuated total reflectance infrared spectroscopy (ATR-FTIR) was used to characterize all the chitin samples extracted from BSFL samples at different growing stages and compare them to a commercial chitin. Thid comparison with a commercial chitin was performed to determine the presence of residual impurities within the material (Figure 3). ATR-FTIR was also used to determine the changes undergone by the samples during each process step; the spectra are reported in the Supplementary Materials for the sake of brevity.

The high-frequency region (4000–3000 cm^−1^) displays bands at (i) 3478 and 3437 cm^−1^ due to two families of hydroxyl groups engaged in intramolecular hydrogen bonds; (ii) at 3261 cm^−1^ due to the stretching of the NH groups of the chitin chains in the form of hydrogen-bonded amide groups; and (iii) at 3102 cm^−1^ due to an overtone of vibrations of amide II at 1560 and 1620 cm^−1^ [43].

In the frequency range between 3000 and 2800 cm^−1^, there are five different bands associated with the symmetrical and asymmetrical stretching of the CH_3_, CH_2_ and CH groups that make up the β-D-glucose units. The bands at 2961 and 2875 cm^−1^ are due to the asymmetrical and symmetrical stretching of the CH_3_ groups, respectively, whereas the corresponding vibrations of the CH_2_ groups are found at 2925 and 2855 cm^−1^; the latter bands are more intense in the spectrum of the BSFL-IL, which is possible because of the presence of lipids not completely removed during the purification stage (see below the ss-NMR data). The band at 2889 cm^−1^ is assigned to the stretching of the CH groups.

In the low-frequency region (1750–400 cm^−1^), the chitin vibrations are present as follows: (i) the NH and CO vibrational modes of the amide groups are between 1660 and 1500 cm^−1^; and (ii) at wavenumbers lower than 1500 cm^−1^, the bands of the CH_3_ and CH_2_ deformation modes are present, along with skeletal collective vibrations. Table 3 displays the band positions of the chitin extracted from BSF-IL and BSFL-EL, and commercial chitin and the values reported in the literature.

### 3.4. Nuclear Magnetic Resonance

Nuclear magnetic resonance is a very common technique used for the characterization of chitin. ^1^H, ^13^C, ^15^N and ^31^P NMR data can provide important information such as the acetylation degree, purity, and allomorphic form [41,42,43].

**Figure 4:**
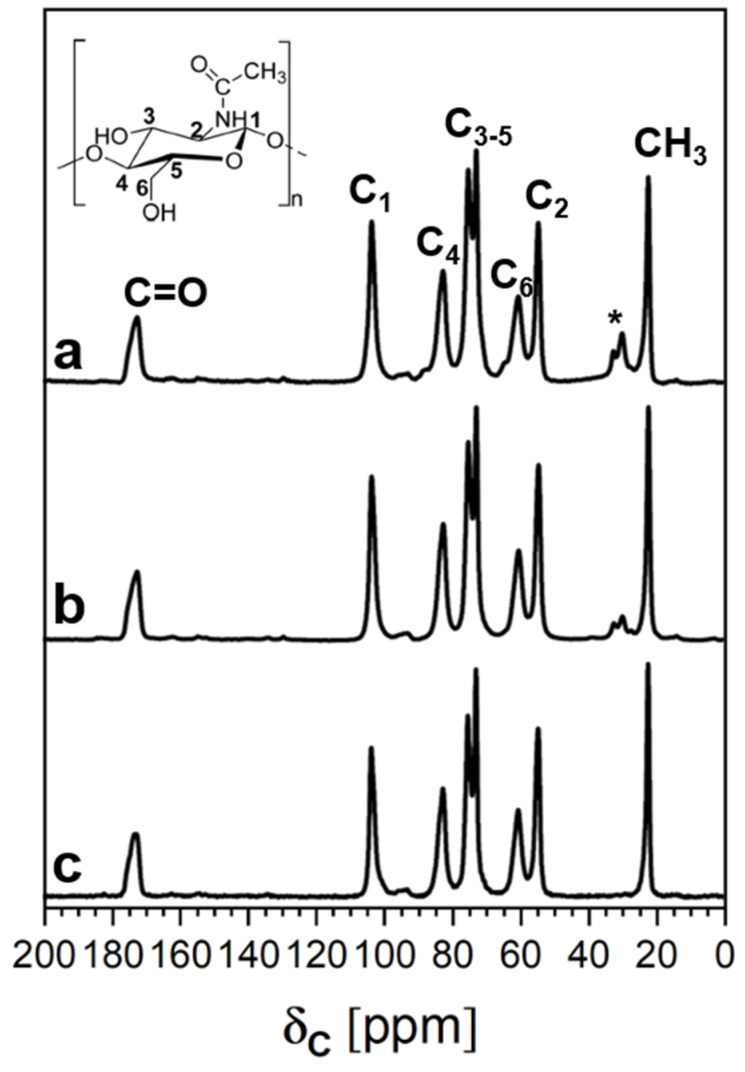
^13^C CPMAS NMR spectra of the extracted chitin at the start and at the end of the BSFL life cycle for BSFL-IL (a) and BSFL-EL (b), respectively, compared with a commercial chitin (c). * ^13^C peaks due to the methylene units of lipids.

The ^13^C CPMAS NMR spectra of the extracted chitin, along with the spectrum of the commercial sample reported for comparison, are shown in Figure 4.

There are no relevant differences between the chitin sample extracted from BSFL at the start or at the end of their life cycle. Both samples show additional peaks in the 27–34 ppm range due to the methylene carbons of residual lipids [46].

For the completeness of the data, the ^13^C CPMAS NMR spectrum of a commercial chitosan, along with the ^1^H MAS NMR spectra of extracted chitin samples and commercial chitin, are provided in the Supplementary Materials.

The degree of acetylation (DA%) could be determined using Equation (2) [47,48]:

(2) DA%=ICH3IC1+IC2+IC3+IC4+IC5+IC66×100

where I_CH3_ is the intensity of the peak of the methyl group and I_Cn_ is the intensity of the peaks due to the six different carbons of the monomeric unit ring. The intensity of the ^13^C CPMAS NMR peaks and the DA% are reported in Table 4.

Both the chitin samples extracted from BSFL and the commercial chitin show a high DA%, reaching a remarkable value of 94% in the case of the samples extracted from BSFL at the end of their life cycle.

Our results suggest that the DA% for chitin samples depends on the chitin source and the method used for the extraction [49].

### 3.5. Thermogravimetric Analysis

**Figure 5:**
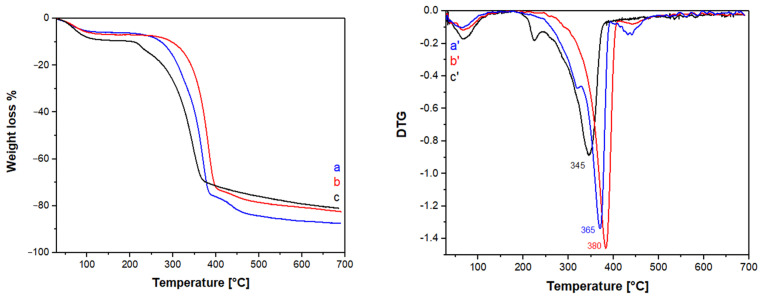
Thermograms (TG) and respective first derivative curves (DTG) of the extracted chitin samples BSFL-IL (a, a′) and BSFL-EL (b, b′), compared with a commercial chitin (c, c′).

Thermogravimetric analysis (TGA) was used to determine the thermal stability of the extracted materials compared with that of a commercial chitin (Figure 5).

Thermogravimetric analyses and the derivative curves (DTG) show the presence of three different degradations in the 30–150, 200–400, and 400–700 °C range, respectively. For all samples, the first weight loss at 30–150 °C is due to the loss of adsorbed water [25,28,50].

The second weight loss at 200–400 °C is due to the thermal degradation of the chitin chains in the samples: deacetylation, dehydration, the loss of amino groups and the cross-linking reactions of chitin chains take place in this temperature range [50,51,52].

The last weight loss at 400–700 °C is due to the depolymerization and degradation of chitin to form volatile molecules with a low molecular weight and char [50,53].

The commercial samples show an additional weight loss at 250 °C of unknown nature. The weight loss % in each temperature range is reported in Table 5.

Both chitin samples extracted from BSFL have a higher degradation temperature, at 365 and 380 °C for BSFL-IL and BSFL-EL, respectively, than the commercial chitin degrading at 345 °C.

## 4. Discussion

Chitin is used for countless applications, such as medical, agricultural, and environmental applications, and in the food and textile industry. It is currently extracted from various animal and plant sources, mainly crab and shrimp shells. However, these natural sources are dependent on seasonality, with moderately long growth times and specific conditions, and all these sources have a high environmental impact. Over the past few years, BSFL have attracted a great deal of attention as a renewable source for various applications, such as the production of biodiesel fuel, animal feeds, and cosmetics [1,11]. BSFL can grow rapidly and on a wide variety of organic materials, and they are extensively used as a means of disposing of household organic waste [1]. The extraction of chitin from BSFL at different stages of life was optimized in this work and the samples obtained were compared to a commercial chitin extracted from arthropod exoskeletons [22,29].

The XRD profiles show the characteristic diffraction peaks of chitin extracted from other animal sources; however, extra phases due to residues from the various process steps are also present. The crystallinity (CrI%) of the chitin extracted from BSFL at the initial life stage and the commercial sample is similar, whereas the chitin extracted from BSFL at the end-of-life stage shows a higher CrI% (at about 78%).

Infrared spectroscopy does not allow the chitin extracted from BSFL samples to be differentiated from that of commercial origin. However, some lipidic impurities are detected in the chitin extracted from BSFL samples, especially at the initial stage of life. This result was confirmed by the NMR spectra, where the signals of methyl and methylene carbons at 27–34 ppm were more intense for the chitin extracted from the BSFL-IL sample (Figure 4, curve a). Moreover, the ^13^C CPMAS NMR spectra show that the DA% increases during the BSFL life cycle, reaching a remarkable value of 94% at the end of the BSFL’s life.

The different CrI%, DA%, and thermal stability of the samples produced in this work suggest that chitin biopolymers with an increased molecular weight and tighter chain packing can be produced depending on the BSFL’s stage of life [19,25,28,53].

## 5. Conclusions

Two different chitin samples were successfully extracted from BSFL at different stages of the life cycle (10 and 20 days after hatching). This work used three process steps to extract substantially purified chitin, namely, the removal of lipids, mineral salts, and protein from BSFL. X-ray diffraction analysis showed that the crystal index (CrI%) of the extracted chitin increases from 71% to 78% during the life of BSFL. A remarkable DA% of 94%, one of the highest reported in the literature, was detected by the ^13^C CPMAS NMR spectra for the extracted sample at the end of the BSFL life cycle (BSFL-EL). This sample also displays the highest thermal stability (380 °C) if compared with the BSFL at their initial stage of life (365 °C) and the commercial sample (345 °C). Moreover, with this method, the yield % of the extracted chitin for BSFL-IL and BSFL-EL was, respectively, 19% and 24%. In the literature, the yield % for chitin extracted from insects is between 5% to 15% (from 18% to 60% for crustaceans) [54].

All these results suggest that chitin with an increased molecular weight and tighter chain packing can be produced by increasing the BSFL life cycle. This enhanced chitin can offer superior mechanical and barrier properties owing to its greater crystallinity, making it ideal for use in food packaging, contributing to better food preservation and the extension of shelf-life. Furthermore, all the data collected suggest that it is possible to obtain chitin with different application-specific properties from BSFL by modulating the days of larval growth.

## Figures and Tables

**Table 1:** Composition (wt. %) of water, lipids, mineral salts, protein, and chitin for the BSFL-IL and BSFL-EL after drying the materials at 50 °C overnight.

Sample	Water	Lipid	Mineral	Protein	Chitin
BSFL-IL	8.0 ± 1.6	23.6 ± 5.3	20.6 ± 3.2	29.7 ± 3.7	18.1 ± 2.0
BSFL-EL	8.2 ± 1.3	21.7 ± 4.9	20.9 ± 2.6	26.9 ± 4.2	22.3 ± 2.3

**Table 2:** Crystal Index (CrI%) of different extracted chitin samples and commercial chitin.

Chitin Source	CrI%
BSFL-IL	71.91 ± 0.13
BSFL-EL	77.84 ± 0.08
Standard Commercial	71.10 ± 0.02

**Table 3:** Peak positions of the FTIR spectra of the extracted chitin compared with those in the literature.

Vibrational Mode	Band Position [cm^−1^]			
	Extracted Chitin	Commercial Chitin	Literature	Reference
CN amide III	1309–1311	1311	1320–1310	[2,28,44,45]
δ CH_3_	1372–1376	1376	1370–1375	[24]
δ + ν NH, CO	1655–1620 1560–1548	1655–1620 1560	1620–1650 1550–1560	[23,25,28,45]
ν CH glucose ring CH_2_OH, CH_3_	2961, 2925, 2855–2889	2875, 2889, 2919, 2925, 2961	3000–2800	[25,28,45]
amide II overtone	3102	3102	3100–3110	[2,24]
ν NH	3261	3261	3255–3270	[2]
ν OH	3478–3437	3478–3437	3280–3450	[2,24,25,28,45]

**Table 4:** ^13^C CPMAS NMR peak intensity and the degree of acetylation (DA%) of different chitin samples.

Chitin Source	Peaks Intensity							DA%
	CH_3_	C1	C2	C3	C4	C5	C6	
BSFL-IL	11.5	15	11	16	13.5	19	14	78
BSFL-EL	13.5	15	14	13	14	18	12.5	94
Commercial	12	13	15	15	14	17	14	82

**Table 5:** Weight loss % at different temperature ranges for the different chitin samples.

	Weight Loss %		
Temperature Range	BSFL-IL	BSFL-EL	Standard Commercial
30–150 °C	5	7	10
200–400 °C	75	72	70
400–700 °C	87	83	81

## Data Availability

The original contributions presented in the study are included in the article/Supplementary Material, further inquiries can be directed to the corresponding author.

## Supplementary Materials

The following supporting information can be downloaded at https://www.mdpi.com/article/10.3390/polym16202861/s1. Figure S1: FTIR spectra of raw sample (a), after the lipid removal step (b), after the demineralization step (c), and the chitin extracted (d) from BSFL-IL samples; Figure S2: FTIR spectra of raw sample (a), after the lipid removal step (b), after the mineral salts removal step (c), and the chitin extracted (d) from BSFL-EL samples; Figure S3: XRPD profiles of extracted chitin from BSFL-IL (a), BSFL-EL (b) and commercial standard (c); Figure S4: ^13^C CPMAS NMR spectra of chitosan standard; Figure S5: ^1^H MAS NMR spectra of chitin extracted samples in the initial stage of life (a), in the end stage of life (b), and in chitin standard (c).
