# Supplementary material for: Chitin Extracted from Black Soldier Fly Larvae at Different Growth Stages
Source: Polymers (Basel). 2024 Oct 10;16(20):2861. doi: 10.3390/polym16202861 (PMC11511002; doi:10.3390/polym16202861)
Supplement: Supplementary file 1 [file polymers-16-02861-s001.zip › polymers-3218121-supplementary.pdf]

Supporting Information:

Chitin extracted from Black Soldier Fly Larvae at different growth stages

Andrea Marangon[1], Geo Paul[2], Riccardo Zaghi[3], Leonardo Marchese[2], Giorgio Gatti[1]

[1] Dipartimento per lo Sviluppo Sostenibile e la Transizione Ecologica, Università degli Studi del Piemonte Orientale, Piazza S. Eusebio 5 - 13100 Vercelli, Italia

[2] Dipartimento di Scienze e Innovazione Tecnologica, Università degli Studi del Piemonte Orientale, Viale Teresa Michel 11 - 15121 Alessandria, Italia

[3] Myia SA, Via Industria 12 - CH - 6710 Biasca, Svizzera

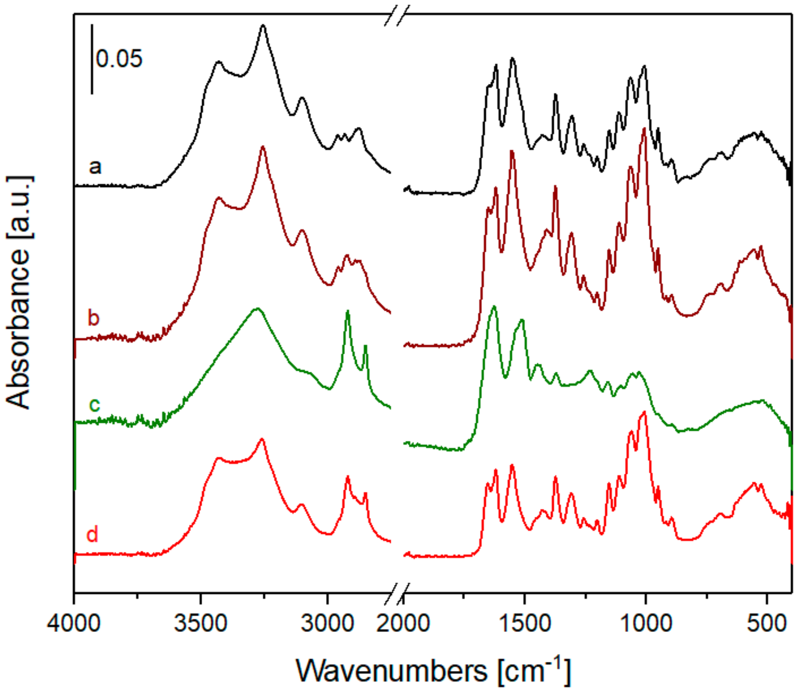

Figure S1: FTIR spectra of raw sample (a), after lipid removal step (b), after demineralization step (c), and extracted chitin (d) of BSFL-IL samples.

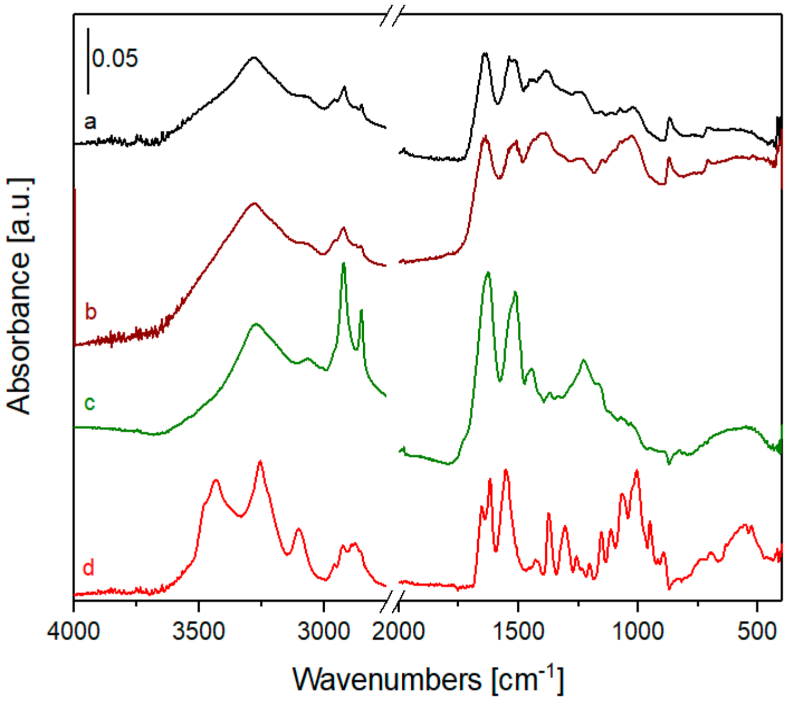

Figure S2: FTIR spectra of raw sample (a), after lipid removal step (b), after mineral salts removal step (c), and extracted chitin (d) of BSFL-EL samples.

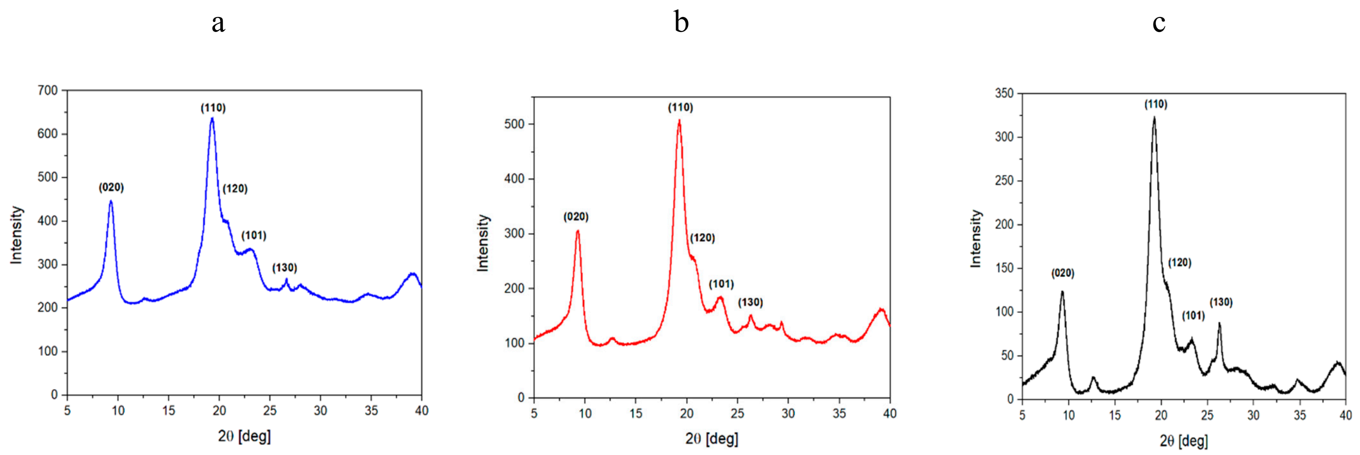

Figure S3: XRPD profiles of extracted chitin from BSFL-IL (a), BSFL-EL (b) and commercial standard (c)

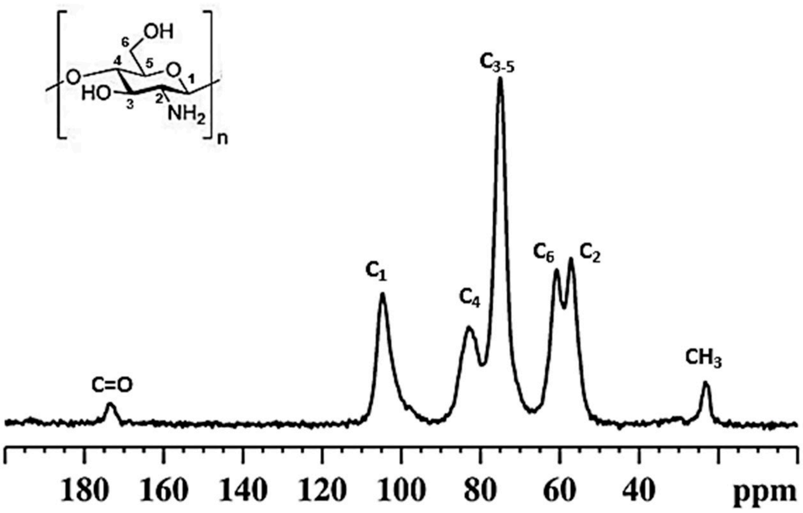

Figure S4: ^{13}C CPMAS NMR spectra of chitosan standard

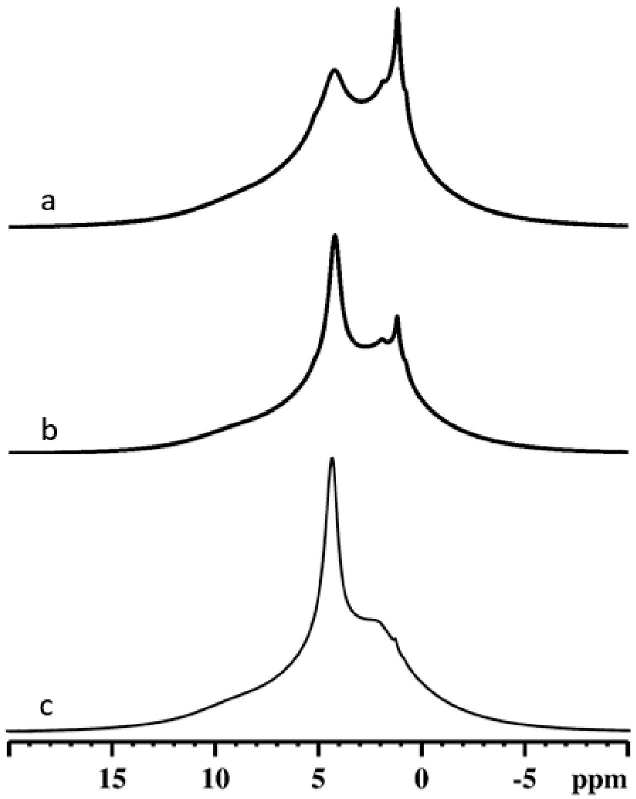

Figure S5: ^{1}H MAS NMR spectra of extracted chitin samples in the initial stage of life (a), in the end stage of life (b), and chitin standard (c)
